# Supplementary material for: Consumer demand for healthy beverages in the hospitality industry: Examining willingness to pay a premium, and barriers to purchase
Source: PLoS One. 2022 May 2;17(5):e0267726. doi: 10.1371/journal.pone.0267726 (PMC9060329; doi:10.1371/journal.pone.0267726)
Supplement: S1 Appendix — (DOCX) [file pone.0267726.s001.docx]

**Appendix 1** Summary statistics of the sample

| **Variable** | **N** | **%** |
| --- | --- | --- |
| *Country* |  |  |
| Australia | 808 | 79.14 |
| New Zealand | 213 | 20.86 |
| *Gender* |  |  |
| Female | 508 | 50 |
| Male | 508 | 50 |
| *Age* |  |  |
| 18-24 | 168 | 16.45 |
| 25-34 | 181 | 17.73 |
| 35-44 | 173 | 16.94 |
| 45-54 | 164 | 16.06 |
| 55-64 | 151 | 14.79 |
| 65 and over | 164 | 16.06 |
| *Relationship Status* |  |  |
| Single | 398 | 39.64 |
| In a relationship | 197 | 19.26 |
| Married | 409 | 40.74 |
| *Employment Status* |  |  |
| Employed full-time | 311 | 30.46 |
| Employed part-time | 221 | 21.65 |
| Unemployed | 67 | 6.56 |
| Student | 68 | 6.66 |
| Retired | 162 | 15.87 |
| Homemaker | 80 | 7.84 |
| Self-employed | 66 | 6.46 |
| Unable to work | 46 | 4.51 |
| *Annual Household Income* |  |  |
| Less than 40K | 233 | 25.27 |
| 40-60K | 223 | 24.19 |
| 61-85K | 151 | 16.38 |
| 86-100K | 121 | 13.12 |
| Over 100K | 194 | 21.04 |
| *Education* |  |  |
| Did not complete high school | 89 | 8.85 |
| Completed high school | 270 | 26.84 |
| Certificate/Diploma | 324 | 32.21 |
| Bachelor degree | 228 | 22.33 |
| Post-graduate degree | 95 | 9.30 |
| Other | 15 | 1.47 |
| *Frequency of Eating Out* |  |  |
| More than 5 times a week | 48 | 4.70 |
| 3-5 times a week | 155 | 15.18 |
| Once or twice a week | 474 | 46.43 |
| Once a month | 195 | 19.10 |
| Only on special occasions | 149 | 14.59 |
| *Spending when Eating Out* |  |  |
| Less than $10 | 61 | 5.97 |
| $10 - $20 | 416 | 40.74 |
| $21 - $35 | 324 | 31.73 |
| $36 - $50 | 144 | 14.10 |
| More than $50 | 76 | 7.44 |
