# Supplementary material for: Consumer demand for healthy beverages in the hospitality industry: Examining willingness to pay a premium, and barriers to purchase
Source: PLoS One. 2022 May 2;17(5):e0267726. doi: 10.1371/journal.pone.0267726 (PMC9060329; doi:10.1371/journal.pone.0267726)
Supplement: S2 Appendix — (DOCX) [file pone.0267726.s002.docx]

**Appendix 2** Chi-square difference tests for age and gender distributions

| **Country** | **Variable** | **Category** | **Observed N** | **Expected N*** | **Chi-square** | **Sig.** |
| --- | --- | --- | --- | --- | --- | --- |
|  |  |  |  |  |  |  |
| Australia | Gender | Male | 398 | 398.9 | 0.003 | 0.998 |
|  |  | Female | 406 | 405.2 |  |  |
|  |  |  |  |  |  |  |
|  | Age | 18-24 | 131 | 100.9 | 10.73 | 0.097 |
|  |  | 25-34 | 147 | 160.3 |  |  |
|  |  | 35-44 | 137 | 142.4 |  |  |
|  |  | 45-54 | 129 | 135.5 |  |  |
|  |  | 55-64 | 120 | 123.7 |  |  |
|  |  | 65+ | 134 | 135.4 |  |  |
|  |  |  |  |  |  |  |
| NZ | Gender | Male | 110 | 104.6 | 0.545 | 0.762 |
|  |  | Female | 102 | 107.4 |  |  |
|  |  |  |  |  |  |  |
|  | Age | 18-24 | 37 | 24.3 | 9.561 | 0.144 |
|  |  | 25-34 | 34 | 36.4 |  |  |
|  |  | 35-44 | 36 | 35.3 |  |  |
|  |  | 45-54 | 35 | 34.2 |  |  |
|  |  | 55-64 | 31 | 31.2 |  |  |
|  |  | 65+ | 30 | 39.6 |  |  |

* Expected N calculated based on Australia and NZ census data.
