# Supplementary material for: Consumer demand for healthy beverages in the hospitality industry: Examining willingness to pay a premium, and barriers to purchase
Source: PLoS One. 2022 May 2;17(5):e0267726. doi: 10.1371/journal.pone.0267726 (PMC9060329; doi:10.1371/journal.pone.0267726)
Supplement: S3 Appendix — (DOCX) [file pone.0267726.s003.docx]

**S3 Appendix : Questionnaire**

**Q1. How often would you eat out in cafes/restaurants or purchase take-away foods? (including delivered meals, UberEats, Deliveroo, etc)**

❐ More than 5 times a week

❐ 3 – 5 times a week

❐ Once or twice a week

❐ Once a month

❐ Only on special occasions

**Q2. Where do you mostly eat out?**

❐ Sit-down Restaurants

❐ Cafés and coffee shops

❐ Fast food and takeaway

❐ Pubs and bars

❐ Food delivery services (e.g. UberEats)

**❐** Other**________________**

**Q3. How much would you typically spend each time you purchase a meal away from home?**

❐ Less than $10

❐ $10 – $20

❐ $21 – $35

❐ $36 – $50

❐ Over $50

**Q4. Who do you mostly go out/eat with? (choose one only)**

❐ Family and Friends

❐ Work colleagues or Business associates

❐ Partner /Spouse

❐ On your own

❐ Other (please specify) ____________________________________

**Q5. What is the most common reason for purchasing meals and drinks away from home? (choose one only)**

❐ Going out with family and friends

❐ Going out with colleagues and business associates

❐ Love to enjoy quality food and wine

❐ Too busy to prepare meals at home

❐ Other (please specify) ____________________________________

**Q6. What meals do you most purchase away from home? (choose one only)**

❐ Breakfast

❐ Lunch on weekdays

❐ Lunch on weekends

❐ Dinner

❐ Other (please specify) ____________________________________

**Q7. Please indicate how frequently you purchase each type of drink when you eat out:**

| Wine | Never | Rarely | Sometimes | Frequently | Every time |
| --- | --- | --- | --- | --- | --- |
| Beer | Never | Rarely | Sometimes | Frequently | Every time |
| Spirits and other alcohol | Never | Rarely | Sometimes | Frequently | Every time |
| Regular soft drinks (with full sugar) | Never | Rarely | Sometimes | Frequently | Every time |
| Diet or low sugar soft drinks | Never | Rarely | Sometimes | Frequently | Every time |
| Probiotic drinks (such as Kombucha and kefir) | Never | Rarely | Sometimes | Frequently | Every time |
| Bottled juices | Never | Rarely | Sometimes | Frequently | Every time |
| Freshly squeezed juices | Never | Rarely | Sometimes | Frequently | Every time |
| Tea (including ice tea) | Never | Rarely | Sometimes | Frequently | Every time |
| Coffee | Never | Rarely | Sometimes | Frequently | Every time |
| Water (including still and sparkling) | Never | Rarely | Sometimes | Frequently | Every time |

**Q8. What drinks/beverages from a restaurant/cafe menu do you consider as being ‘healthy’?**

**____________________________________________________**

**Q9. Why do you consider these drinks as ‘healthy’? (E.g. low sugar, health-star rating, no preservatives, added vitamins and minerals, etc.)**

**____________________________________________________**

**Q10. What are some of the obstacles that restrict you from ordering healthier drinks? (E.g. price, taste, ingredients, unproven health benefits)**

❐ Healthy drinks cost more

❐ Healthy drinks do not taste as good

❐ There are limited options available to purchase

❐ I am not convinced that ‘healthy drinks’ are truly healthy

❐ I am not familiar with the brands that make healthy drinks

❐ Other______________________________

**Q11. The following question is about your willingness to pay for healthy (vs unhealthy) drinks.**

**Suppose that the price of an ‘unhealthy’ drink (i.e. high in sugar, artificial ingredients) from a café/restaurant/takeaway is $5.**

**Now, consider an alternative drink that was healthier (i.e. low in sugar, organic, has proven health benefits etc.), what price would you be willing to pay for the healthy drink?**

| **Healthy Drink Price $** | **Willingness to purchase** | | |
| --- | --- | --- | --- |
| $10 | I would never buy | I might buy occasionally | I would buy most of the time |
| $9 | I would never buy | I might buy occasionally | I would buy most of the time |
| $8 | I would never buy | I might buy occasionally | I would buy most of the time |
| $7 | I would never buy | I might buy occasionally | I would buy most of the time |
| $6 | I would never buy | I might buy occasionally | I would buy most of the time |
| $5 | I would never buy | I might buy occasionally | I would buy most of the time |

**Q12. The following set of questions relate to your lifestyle and attitudes toward food and healthy eating. Please respond to each question showing your level of agreement (1= Strongly Disagree to 7 = Strongly Agree).**

| Healthy eating is something I do frequently | Strongly Disagree | Disagree | Slightly Disagree | Undecided | Slightly Agree | Agree | Strongly Agree |
| --- | --- | --- | --- | --- | --- | --- | --- |
| Healthy eating is something I find hard to do | Strongly Disagree | Disagree | Slightly Disagree | Undecided | Slightly Agree | Agree | Strongly Agree |
| Healthy eating is something that is part of my normal routine | Strongly Disagree | Disagree | Slightly Disagree | Undecided | Slightly Agree | Agree | Strongly Agree |
| Healthy eating is something I do automatically/without having to think consciously about it | Strongly Disagree | Disagree | Slightly Disagree | Undecided | Slightly Agree | Agree | Strongly Agree |
| It’s important to me that the food I eat on a typical day contains vitamins and minerals | Strongly Disagree | Disagree | Slightly Disagree | Undecided | Slightly Agree | Agree | Strongly Agree |
| It’s important to me that the food I eat on a typical day is good for my appearance | Strongly Disagree | Disagree | Slightly Disagree | Undecided | Slightly Agree | Agree | Strongly Agree |
| It’s important to me that the food I eat on a typical day is nutritious | Strongly Disagree | Disagree | Slightly Disagree | Undecided | Slightly Agree | Agree | Strongly Agree |
| I eat what I like and do not worry about the healthiness of food | Strongly Disagree | Disagree | Slightly Disagree | Undecided | Slightly Agree | Agree | Strongly Agree |
| The healthiness of a food has little impact on my food choices | Strongly Disagree | Disagree | Slightly Disagree | Undecided | Slightly Agree | Agree | Strongly Agree |
| I think of myself as someone who generally thinks carefully about the quality of foods I select | Strongly Disagree | Disagree | Slightly Disagree | Undecided | Slightly Agree | Agree | Strongly Agree |
| I think of myself as someone who generally thinks carefully about the health consequences of my food choices | Strongly Disagree | Disagree | Slightly Disagree | Undecided | Slightly Agree | Agree | Strongly Agree |
| I think of myself as the sort of person who is concerned about the long-term effects of my food choices | Strongly Disagree | Disagree | Slightly Disagree | Undecided | Slightly Agree | Agree | Strongly Agree |
| It’s important to me that the food I eat on a typical day is not expensive | Strongly Disagree | Disagree | Slightly Disagree | Undecided | Slightly Agree | Agree | Strongly Agree |
| It’s important to me that the food I eat on a typical day is cheap | Strongly Disagree | Disagree | Slightly Disagree | Undecided | Slightly Agree | Agree | Strongly Agree |
| I am willing to pay more for food and drinks that have proven health benefits | Strongly Disagree | Disagree | Slightly Disagree | Undecided | Slightly Agree | Agree | Strongly Agree |

**Q13. The following questions are about your attitudes to purchasing healthy when eating out. Please respond to each question showing your level of agreement (1= Strongly Disagree to 7 = Strongly Agree).**

| I would purchase healthy drinks if there was more variety of products in the market | Strongly Disagree | Disagree | Slightly Disagree | Undecided | Slightly Agree | Agree | Strongly Agree |
| --- | --- | --- | --- | --- | --- | --- | --- |
| I would purchase healthy drinks if there was more information about their health benefits | Strongly Disagree | Disagree | Slightly Disagree | Undecided | Slightly Agree | Agree | Strongly Agree |
| I would purchase healthy drinks if the product had a strong brand | Strongly Disagree | Disagree | Slightly Disagree | Undecided | Slightly Agree | Agree | Strongly Agree |
| I would purchase healthy drinks if the product had a strong social media following | Strongly Disagree | Disagree | Slightly Disagree | Undecided | Slightly Agree | Agree | Strongly Agree |
| I would purchase healthy drinks if the product was natural and organic | Strongly Disagree | Disagree | Slightly Disagree | Undecided | Slightly Agree | Agree | Strongly Agree |
| I would purchase healthy drinks if the products were locally made | Strongly Disagree | Disagree | Slightly Disagree | Undecided | Slightly Agree | Agree | Strongly Agree |
| I would purchase healthy drinks if the beverages had proven health benefits | Strongly Disagree | Disagree | Slightly Disagree | Undecided | Slightly Agree | Agree | Strongly Agree |
| I would purchase healthy drinks if there were more choice of brands | Strongly Disagree | Disagree | Slightly Disagree | Undecided | Slightly Agree | Agree | Strongly Agree |
| I would purchase healthy drinks if the drinks product could be available on-tap | Strongly Disagree | Disagree | Slightly Disagree | Undecided | Slightly Agree | Agree | Strongly Agree |
| I would purchase healthy drinks if the product had a health star rating on its label. | Strongly Disagree | Disagree | Slightly Disagree | Undecided | Slightly Agree | Agree | Strongly Agree |

**Q14. Gender (please circle one):** Male / Female **/** Other

**Q15. Age**

❐18 – 24 years

❐ 25 – 34

❐ 35 – 44

❐ 45 – 54

❐ 55 -64

❐ 65+

**Q16. What is the highest level of education that you have completed?**

❐ Did not complete high school

❐Completed high school

❐Certificate / Diploma

❐Bachelor’s Degree

❐Post-Graduate Degree

❐Other (please specify) _______________________

**Q17. What is your usual occupation?: _____________________________________________**

**Q18. What is your postcode?: __________________**

**Q19. What is your current relationship status?:**

❐ Single

❐ In a relationship (but not currently married)

❐ Married

**Q20. Do you have children?:**

❐ No

❐ Yes, I have __________ child/children

**Q21. What is your current employment status?:**

❐ Employed full time (40 or more hours per week)

❐ Employed part time (up to 39 hours per week)

❐ Unemployed

❐ Student

❐ Retired

❐ Homemaker

❐ Self-employed

❐ Unable to work

**Q22: What is your annual household income?:**

❐ Less than $40 000

❐ $40 000 – $60 000

❐ $61 000 – $85 000

❐ $86 000 – $100 000

❐ Over $100 000

❐ Do not wish to disclose
