# Supplementary material for: Consumer demand for healthy beverages in the hospitality industry: Examining willingness to pay a premium, and barriers to purchase
Source: PLoS One. 2022 May 2;17(5):e0267726. doi: 10.1371/journal.pone.0267726 (PMC9060329; doi:10.1371/journal.pone.0267726)
Supplement: Attachment — Submitted filename: RH_2 Dec_PLOS_ResponsetoReviewer.docx [file pone.0267726.s005.docx]

**Consumer demand for healthy beverages in the hospitality industry: Examining willingness to pay a premium, and barriers to purchase**

**Response to Reviewer Comments:**

**Reviewer 1:**

**Point 1.1:** The authors offer very interesting insight into consumers’ conceptualisation of healthy beverages and into their willingness to pay a little extra compared to unhealthier drink options. The writing style is clear and elegant and some findings are clearly interesting; however, this manuscript does not present a body of evidence that clearly advances the literature.

**Response 1.1:**

Thank you for your feedback and for raising these concerns. The manuscript has been substantially revised to strengthen its originality and contribution to the body of knowledge. Changes in the manuscript have been highlighted throughout.

We have carefully dissected all your comments and identified 22 points for revisions. The table below identifies each of your comments and provides a response. Wherever possible, we revised the manuscript to address each of your points.

**Point 1.2:** The six hypotheses asks questions that the authors already answered in the Introduction; there are also some issues in regards to some methodological choices and the discussion does not offer new significant insight. I will now explore these issue in more detail below.

**Response 1.2:**

Thank you. The revised manuscript includes substantial improvements to the Introduction, Literature Review, and Discussion sections.

**Point 1.3**: Overall, the paper is very well-written but it is repetitive in quite a few places, for example (a) lines 60-62 on p.3, (b) early on p.6 and (c) lines 337-341 on p.16. In other places, the manuscript presents some relatively or uninformative elements, for example (d) lines 106-111 on p.5, (e) lines 482-489 on p.29, and (f) lines 569-583 on p.33. Relatedly, the Hypotheses Tests presented at Table 6 should be integrated within the text in an effort to streamline the manuscript.

**Response 1.3:**

We have deleted or revised the content on

a) lines 60-62 on p.3,

(b) early on p.6 and

c) lines 337-341 on p.16.

In other places, the manuscript presents some relatively or uninformative elements, for example (d) lines 106-111 on p.5, (e) lines 482-489 on p.29, and (f) lines 569-583 on p.33

This content has been revised or deleted.

(e) lines 482-489

This paragraph has been deleted

and (f) lines 569-583 on p.33.

This content has been revised but not deleted.

Hypotheses Tests presented at Table 6 should be integrated within the text in an effort to streamline the manuscript

Thank you for raising this issue and your recommendation for integrating Table 6 in the text. While this is something that is relatively easy to do, we respectfully request to keep the hypotheses listed in H6 for two main reasons: 1) the manuscript is rich in text at over 11 000 words long and we believe presenting the hypotheses results in H6 to be of benefit to readers interested in accessing the key findings. 2) In our field of study, hypotheses test results are commonly summarised in table format so we would like to stick with this common practice.

**Point 1.4:** As mentioned above, all 6 hypotheses do not seem to explore original questions as the literature the authors discuss already addressed these and thus no incremental value can be easily ascertained.

**Response 1.4:**

Thank you for raising these points and the need to strengthen the originality of the hypotheses. In consideration of the broader feedback around the manuscript’s objectives, positioning, and contribution, we have made substantial revisions throughout the paper including a stronger substantiation for the hypotheses and their value in contributing to new knowledge. Specifically, we have expanded our literature review to include literature from different contexts. This highlights the variances in previous findings and the need to further examine consumer behaviour in regard to healthy beverage purchasing in the context of the hospitality and food service environments. We provide evidence that consumer motivations, attitudes, and behaviours to health products can differ, and these variances due to different context/environment are expressed in the manuscript.

New references added include:

A. Tabassum, J. Ali. Factors Affecting the Consumers’ Willingness to Pay for Health and Wellness Food Products. J. of Agriculture and Food Research 2 (2020): 100076. <https://doi.org/10.1016/j.jafr.2020.100076>.

A. Krystallis, G. Chryssohoidis. Consumers' willingness to pay for organic food: factors that affect it and variation per organic product type Br. Food J., 107 (5) (2005), pp. 320-343

M.L. Loureiro, J.J. McCluskey, R.C. Mittelhammer. Will consumers pay a premium for eco-labeled apples? J. Consum. Aff., 36 (2) (2002), pp. 203-219

O.A. Ogbeide, C. Ford, Stringer, R Consumer Willingness to Pay a Premium for the Health Benefits of Organic Wine. Mayfair Journal of Agribusiness Management. 1 (2015). pp. 1-23.

Z. Gao, T. Schroeder, Effects of label information on consumer willingness-to-pay for food attributes, American Journal of Agricultural Economics, 91 (3) (2006), pp. 795-809.

**Point 1.5:** As a related example, on p.8 (line 176) the authors state that “there are myriad of factors that may contribute…” but then to do not explore what these factors are; it is therefore arduous to appreciate what novel questions and knowledge are offered in the manuscript

**Response 1.5:**

Thank you raising this issue. In rewriting the literature review section of this study, this sentence has been deleted as it does not specifically relate to the research objectives or hypotheses.

**Point 1.6:** The Method is a well-presented section, although a few choices should be further justifies. For example, it is not entirely clear why only consumers who eat out are included in this study; after all, selection of healthy (vs. unhealthier) drinks can be made in supermarkets, too – and, overall, it is likely to represent a greater issue in terms of public’s well-being.

**Response 1.6:**

Thank you form your feedback. In addressing these comments, we have revised both the Introductions and Methods sections. In both these sections make explicit that the objectives and focus of the study is on consumer behaviour in a hospitality industry context, thus the justification for our research scope and sample frame.

**Point 1.7:** There is no justification (e.g., power analysis) for the sample size tested in this study.

**Response 1.7:**

Thank you for the feedback. As per your suggestion we have performed power analysis for our sample size of 885 observations (based on N for Model 5). The post-hoc power test for N=885 is 1, which strongly justifies the sample size for our analysis. We also calculated the minimum sample size needed using power analysis for our empirical analysis and found the minimum number to be 248 observations. Our sample size is around 3.5 times greater than this number. We have added information on our power analysis results in our Results.

**Point 1.8:** A rationale and more in-depth justification should be offered in terms of the selection of items for the three predictors; for example, it is unclear whether the items were selected from previously utilised (and reliable) measures or not. Although the authors offer some reassurance through factor analysis, other types of validity are not discussed nor addressed.

**Response 1.8:**

Thank you for raising this point. Our section ‘Predictor Variables’ under Methods explains the selection of independent variables.

In addition to demographic and behavioral variables, the research instrument captured consumers’ psychological variables relating to attitudes and goals toward health and nutrition, as these goals are hypothesized to influence willingness to pay. These include validated scales from the consumer psychology literature that capture ‘healthy eating goals’, ‘food hedonism goals’ and ‘food economizing goals’.

**Source:**

M.B. McCarthy, A.M. Collins, S.J. Flaherty, S.M. McCarthy Healthy eating habit: A role for goals, identity, and self-control? Psychol Mark 34 (2017) pp. 772-785.

A. Steptoe, T.M. Pollard, J. Wardle Development of a measure of the motives underlying the selection of food: The food choice questionnaire. Appetite 25 (1995) pp. 167-284.

**We report on the results of the factor analysis in Results:**

The review of the literature identified demographic and motivational variables as predictors of consumers’ purchase behavior toward healthy food and beverage products. Motivational factors include Healthy Eating Goals (3 items), Food Economizing Goals (2 items), and Food Hedonism Goals (2 items). Principal component analysis with oblique (Oblimin) rotation was conducted on the seven observed variables. The results of the PCA supports a three-factor solution (eigenvalues > 1) as the best fit for the data and explains 78.9% of the variance in the model. The component matrix shows each observed item loads well onto its corresponding factor. The Cronbach’s alpha for each factor is >.7, supporting scale reliability (internal consistency) (Table 4).

**Table 4.** **Factor analysis results**

| Variable | Item Description | Mean (SD) | Factor 1 | Factor 2 | Factor 3 |
| --- | --- | --- | --- | --- | --- |
| Healthy Eating Goals 1 | It’s important to me that the food I eat on a typical day contains vitamins and minerals’ | 4.7 (1.539) | .858 |  |  |
| Healthy Eating Goals 2 | It’s important to me that the food I eat on a typical day is good for my appearance | 4.24 (1.587) | .757 |  |  |
| Healthy Eating Goals 3 | It’s important to me that the food I eat on a typical day is nutritious | 5.08 (1.399) | .825 |  |  |
| Food Economizing Goal 1 | It’s important to me that the food I eat on a typical day is not expensive | 4.92 (1.369) |  | .751 |  |
| Food Economizing Goal 2 | It’s important to me that the food I eat on a typical day is cheap | 4.41(1.527) |  | .788 |  |
| Food Hedonism Goal 1 | I eat what I like and do not worry about the healthiness of food | 4.19(1.668) |  |  | .461 |
| Food Hedonism Goal 2 | The healthiness of a food has little impact on my food choices | 4.22(1.642) |  |  | .519 |
| *Eigenvalue* |  |  | 2.505 | 1.971 | 1.054 |
| *Total variance Explained* |  |  | 35.789 | 28.153 | 15.052 |
| *Cronbach Alpha* |  |  | .833 | .753 | .769 |

*NOTE: Factor1 = Healthy Eating Goals, Factor 2= Food Economizing Goals. Factor 3= Food Hedonism Goals. Total variance explained = 78.994%*

**Point 1.9:** There are further issues with the selection of the items. The authors state that their strategy was not to mention what a healthy drink is in order to avoid constraining participants’ perceptions. However, items in the Healthy Eating Goals measure indicate specific elements (e.g., containing vitamins and minerals, being good for appearance, being nutritious) that can prime participants in their answers. Other elements that should be addressed is whether foods that are good for the appearance (item for the Healthy Eating Goals measure) are necessarily healthy. It is also somewhat surprising that the criterion variable (WTPP) is about drinks but the predictor measures regard food

**Response 1.9**

Thank you for raising these issues. We will now attempt to explains our rationale.

1. In regard to ‘not to mention what a healthy drink is in order to avoid constraining participants’ perceptions’, this method was to gain insights on ‘healthy’ from a consumers’ perspective, and to address RQ1 and RQ2 from the study RQ1: Which beverages that are sold in hospitality businesses do consumers consider to be ‘healthy’? RQ2: What criteria do consumers use to determine whether a beverage is considered to be ‘healthy’?

Research conducted from nutrition or health sciences perspective adopts an objective assessment of ‘health’. This study is adopts a consumer behaviour framework. The approach to adopt a self-reported assessment of health was adapted from:

R.S. Newson, R. van der Maas, A. Beijersbergen, L. Carlson, C. Rosenbloom International consumer insights into the desires and barriers of diners in choosing healthy restaurant meals. Food Qual Prefer 43 (2015) pp. 63-70.

The items to motivational goals (health eating, economizing, hedonism) were drawn validated scales from:

M.B. McCarthy, A.M. Collins, S.J. Flaherty, S.M. McCarthy Healthy eating habit: A role for goals, identity, and self-control? Psychol Mark 34 (2017) pp. 772-785.

A. Steptoe, T.M. Pollard, J. Wardle Development of a measure of the motives underlying the selection of food: The food choice questionnaire. Appetite 25 (1995) pp. 167-284.

These scales capture food goals that relate to consumptions of food and drinks. They are associated with overall motivations to consumption (eating and drinking). As these were validated scales, we chose not to change the items and to keep as they were validated.

The predictor variables capture psychological factors related to consumers (goals/motivations) in regard to consumption. The criterion WTPP, although specific to beverages, is analysed in the study as the dependence variable in order to examine examines the extent to which these psychographic characteristics of consumers are related to behavioural intentions (i.e. intentions to purchasing at a premium price). The proposed relationships between motivational goals and WTPP are explained in the revised Literature Review.

**Point 1.10:** Further explanations could be offered about the WTPP, too (e.g., the choice of $5 as the comparison point). It is also not entirely clear how the choice data (i.e., the unhealthy $5 vs. the alternative) has been combined with the frequency data (“never”, “occasionally”, or “most of the time”) in order to categorise participants. Is the switch point determine when a participant switches from “never” to “occasionally” or from the latter to “most of the times”? The mention of the “maximum” price that participants were willing to pay (p.13) does not elucidate this issue

**Response 1.10**

Thank you for pointing these out. On average, regular soft drinks in hospitality venues (bar, restaurant, café, etc.) in Australia cost around $4.5 - $5, which has guided our choice of $5 as the comparison point. We have added content in the section on Criterion variable to further explain:

*The reference point of $5 was selected as it is the average price of a typical sugar sweetened beverage sold in hospitality venues in Australia and New Zealand. The overall prices of food and beverages in the two countries are on par, in addition, the same scale was used for both countries as the AUD and NZD currencies are* *of approximately equal value (1 AUD = 1.07 NZD).*

Regarding the switch point for the WTPP variable, for each price point we have identified a respondent as willing to spend that specific amount if his/her response was a willingness to pay – this included “occasionally” or “most of the time”. Similarly, if the response was “I would never buy”, we have categorised it as the respondent is not willing to pay that specific amount for the healthy beverage. In other terms, the switch point was from “never buy” to “buy”. We have amended the Criterion variable section to include this description.

**Point 1.11:** In terms of the Control variables, it is unclear why Country has been included and why would the authors expect differences between AUS and NZ. The same question arises when employment and marital status and having children are included in the analyses, too. Whilst censored regression seems a reasonable choice, it is not clear why the authors have not chosen a more straightforward alternative such as ordinal regression, which could be appropriate for this data set, too

**Response 1.11:**

Thank you for the feedback. Based on our review of literature, demographic characteristics such as the country of residence, employment status, income, ag etc. are likely to affect how consumers view healthy beverages and thus their willingness to pay (or pay a premium). Moreover, these variables are also likely to affect (correlated with) our main variables of interest; healthy eating goals, food economizing goals, and the food hedonism goals. Thus, not including the abovementioned control variables could lead to omitted variable bias and influence the coefficients we obtain for the main variables of interest. As a result, we include them as control variables in our empirical analysis so as not to bias the main findings of our paper.

Regarding your point about censored vs ordinal regression, considering the design of our dependent variable we believe a count data model (Poisson) is a better choice for several reasons. Ordinal regression analysis is suitable for when certain outcomes are “higher/better” or “lower/worse” than others, but it does not really matter by how much. On the other hand, when the dependent variable has non-negative integers and if these integers have a “meaning” (for instance a value of “4” represents a quantity two times more than “2”), a count data model is considered more appropriate (and informative). Using the Poisson model, we are also able to control for the “top-coding” issue as mentioned in the manuscript.

Our section on ‘Model description and analysis techniques’ presents the following explanation:

*Data were analyzed through SPSS and STATA. Descriptive analysis, factor analysis, and censored Poisson regression were used. Our dependent variable (WTPP), as shown in Table 1, is composed of nonnegative integers ranging from 0 to 6. Given the count data nature of the criterion variable, we use the Poisson regression method. The distribution of count data is discrete (not continuous) and it is limited to nonnegative values. Thus, the main assumption of normal distribution in a linear regression analysis (such as ordinary least squares – OLS) is likely to be violated. The OLS would also produce negative predicted values, which are impossible. For these reasons, the use of a nonlinear estimation model of Poisson is preferred [73,74].*

*In addition, considering that the criterion variable is top censored (i.e. at $10), respondents willing to pay $10 may also be willing to pay a higher amount (e.g. $11, $12*, $13, etc.). *Since their willingness to pay an amount higher that $10 is not captured in our survey design, we analyze our data using censored regression to take the top coding into consideration [74]. Below, we formally present our censored Poisson regression analysis*.

**Point 1.12:** It is also not accessible to the reader what the purpose of the model and the equations presented on pp.14-16. In their current form, these considerations seem to refer to general formulations of the Poisson regression analysis that therefore offer no information that is specific to this manuscript and its analysis. Are the authors justifying the selection of a particular log likelihood function that has been amended on the basis of the purpose of this specific analysis? If the equations and considerations are general and bear no identifying information for the specifics of the present analysis, they could be omitted

**Response 1.12:**

Thank you for the feedback. While the beginning of this section refers to general formulation of the Poisson regression analysis, we introduce the censoring to the general formulations, leading us to present the censored Poisson model examined in our study. We believe explicitly presenting the model would help the reader to see where the censoring is originating from and how it is altering a general Poisson regression analysis.

*Below, we formally present our censored Poisson regression analysis.*

*For the Poisson model, the probability mass function is the Poisson distribution:*

$$\Pr\left( Y=y \right)=\frac{e^{-\mu}\mu^{y}}{y!}, y=0,1,2,\ldots$$

*With the following exponential mean parametrization:*

$$\mu_{i}=exp\left( x_{i}^{'}\beta\right), i=1,2,3,\ldots,N$$

*Thus, we end up with the following probability function:*

$$f\left( y_{i};\mu_{i} \right)=\frac{e^{-\mu_{i}}{\mu_{i}}^{y_{i}}}{y_{i}!}, i=1,2,\ldots, n$$

*Where* $\mu_{i}=exp\left( x_{i}\beta\right)$*,* $x_{i}$ *is a vector of exogenous variables and* $\beta$ *is a vector of unknown parameters. These unknown parameters are the regression coefficients that are estimated from a dataset using the method of maximum likelihood.*

*Since we do not observe all* $y_{i}$ *exactly, we introduce a censored Poisson model. To this end, assume the true* $y_{i}^{*}$ *is observed only below a censoring point* $c_{i}$*. Thus, we have the following:*

$$y_{i}=\left\{ \begin{aligned} y_{i}^{*}, if y_{i}^{*}<c_{i} \\ c_{i}, if y_{i}^{*}\geq c_{i} \end{aligned} \right.$$

*In our case, the data are censored from above at the value of 6 because of top-coding. In other words, for us* $c_{i}=6.$ *Ignoring the censoring would lead to inconsistent parameter estimates.*

*If* $y_{i}$ *is censored, we know that:*

$Pr\left( y_{i}\geq c_{i} \right)=\sum_{j=c_{i}}^{\infty} Pr\left( y_{i}=j \right)=\sum_{j=c_{i}}^{\infty} f(j)=1-\sum_{j=0}^{c_{i}-1} f\left( j \right)=1-F(c_{i}-1)$

*To write down the maximum likelihood estimator (MLE), it is convenient to introduce an indicator variable* $d_{i}$ *such that:*

$$d_{i}=\left\{ \begin{aligned} 1, if y_{i}^{*}\geq c_{i} \\ 0, otherwise \end{aligned} \right.$$

*Then, the log likelihood function can be written as:*

$$\mathcal{L}\left( \beta\right)=\sum_{i=1}^{n} \left[ \left( 1-d_{i} \right)logf\left( y_{i} \right)+d_{i}log\left\{ 1-F\left( c_{i}-1 \right) \right\} \right]$$

*The estimation is by maximum likelihood and the initial values are taken from the Poisson model.*

Our preference it to maintain the formulas and explanation in the body of the manuscript, however, if requested we could consider moving this to an Appendix,

**Point 1.13:** The data stemming from the open questions, due to issues of non-independence, are not analysed inferentially and thus conclusions drawn on descriptive analyses should be offered more tentatively, which weakens the manuscript. Further details would benefit the manuscript as well. For example, on Table 2 the total count is 1,373 responses and thus a breakdown of whether all participants provided at least a response and how many offered multiple responses, and how many of them could be presented. Table 3 offers half as many total responses (776) so it is clear that the rate of non-responding was higher for this item. The same comment applies to the data summarised on Table 7.

**Response 1.13:**

Thank you for your feedback. There are a couple of parts to this comment so we will attempt to address them individually.

The first point regarding **“**conclusions drawn on descriptive analyses should be offered more tentatively” is well noted. Our research questions and data analysis consider both descriptive statistics (reported at the sample level), as well as inferential statistics.

Regarding RQ1: Which beverages that are sold in hospitality businesses do consumers consider to be ‘healthy’? RQ2: What criteria do consumers use to determine whether a beverage is considered to be ‘healthy’? We note that these exploratory questions, and the manners in which data is collected and analysed, represent responses specific to our sample and not to the broader population. Our revised manuscript acknowledges that we present our results as being specific to our sample. For example, in the Discussion we report “*Our findings, at least at the sample level, suggest a growing acceptance by hospitality consumers of the taste and higher prices associated with helathier beverage options. Of course, there will be a segment of the market who remain skepticaly toward the true healthiness of ‘healthy’ beverage products; as is the case in our sample where 21% of respondents reported a major barrier to purchasing was that they were unconvinced the products were truly healthy.*

We also amended the limitations to include *“Our insights on what consumers determine to be healthy beverages and their attributes are based on a descriptive analysis of the sample response, thus, the generalizability of these findings to the broader population needs further testing. Nevertheless, its is important to note that out sampling design was representative of the broader population for each country. We encourage future studies to collect data from different countries and examine cross-cultural/country differences among consumers’ purchase behaviors.”*

However, while the findings related to perceptions of health drinks and the attributes are based on sample data, our sampling method was representative of the population of interest:

*Data were collected from 1021 consumers across Australia (N=808, 79.14%) and NZ (N=213, 20.86%) (S1 Appendix). There was an even spread of male and female respondents, and a relatively even spread of respondents across the six age categories measured (ranging from 18 to 65 and over). Most respondents were in full- or part time work and the largest income group was those earning less than $40K in annual household income (25.27%), followed by households earning between $40-$60K annually (24.19%). Education wise, the largest group were those with a Certificate/Diploma (32.21%), followed by those who completed high school (26.84%), and those with a Bachelor degree (22.33%). To assess the representativeness of our sample, we compared the distribution of our Australia and NZ samples’ gender and age distributions to their respective country’s census data using chi square difference tests. These tests produced non-significant results (S2 Appendix), indicating that the gender and age distributions of our sample to be similar to the general population of Australia and NZ, supporting the representativeness of our sample.*

In regard to the number of responses from the open-ended questions (as reported in Table 2 and Table 3), this is simply a count of the number of times a response was reported. For example, for the survey question “What drinks/beverages from a restaurant/cafe menu do you consider as being ‘healthy’?” the response of ‘Tea’ appeared on 158 occasions in our data set. Research participants were free to report multiple beverage products, and we counted the number of times a product had been reported by the sample.

A similar approach was used for Table 3 where we categorised responses. The main attributes that were reported related to sugar, being natural and free of artificial additives, include vitamins and minerals (as we presented in Table 3). The majority of respondents reported more than a single attribute, as expected from an open-ended question. We are interested in exploring, from a consumer perspective, the multiple ranges of attributes that they can recall as being characteristics of health beverages. This is what we report and addresses in RQ1 and RQ2 of the study. The results in table 7 are also based on an open category question were respondents could give multiple answers. We have now updated table 7 to include the ‘Other’ responses (n=83). However, we do not go into detail in analysing the ‘Other’ category responses as most respondents did not specify, or reported unusable responses such a ‘I like alcohol’, or ‘water is free’ etc.

**Point 1.14:** In hierarchical regression models, usually the predictors of interest (in this case food hedonism, economising and healthy eating goals) are entered in the last step(s) in order to explore whether they can explain any residual variance that is not accounted for by known or control predictors. Why is it the other way around here?

**Response 1.14:**

Thank you for pointing this out. As more commonly done in our field of research, we have included the variables of interest starting from Model 1. The idea behind it is to observe if including other variables (control variables) in later models changes the coefficients and significance of our predictors of interest. If the estimates for these variables do not show much variation across models, that would suggest robustness in our findings on how these variables of interest are affecting our dependent variable.

We acknowledge your comments regarding the hierarchal regression procedures; thus, we have re-estimated the models in the order that you suggested. Below please find the table following the hierarchical regression. Note that Model 4 and 5, which are the most informative models in our study they test our hypotheses related to the motivational goals and WTPP, have not changed. Thus, we have chosen to keep table 5 in the same order in our paper.

**Response to Reviewer Comment 1.14**

**Censored Poisson regression results for the determining factors of Willingness to Pay a Premium (WTPP) – Hierarchical Regression Models**

| **Variable** | **Model 1** | **Model 2** | **Model 3** | **Model 4** | **Model 5** |
| --- | --- | --- | --- | --- | --- |
| *Age* (Base: 65 or older) | | | | | |
| - 18 to 24 | **0.506***** | **0.357***** | **0.307***** | **0.456***** | **0.471***** |
|  | (0.0610) | (0.0978) | (0.0993) | (0.101) | (0.101) |
|  |  |  |  |  |  |
| - 25 to 34 | **0.394***** | **0.170*** | 0.118 | **0.238**** | **0.248**** |
|  | (0.0609) | (0.0936) | (0.0949) | (0.0960) | (0.0962) |
|  |  |  |  |  |  |
| - 35 to 44 | **0.258***** | 0.0157 | -0.00977 | 0.0871 | 0.0991 |
|  | (0.0627) | (0.0919) | (0.0928) | (0.0935) | (0.0940) |
|  |  |  |  |  |  |
| - 45 to 54 | **0.112*** | -0.113 | -0.119 | -0.0504 | -0.0451 |
|  | (0.0649) | (0.0926) | (0.0934) | (0.0936) | (0.0939) |
|  |  |  |  |  |  |
| - 55 to 64 | -0.0437 | **-0.195**** | **-0.190**** | -0.129 | -0.125 |
|  | (0.0685) | (0.0873) | (0.0876) | (0.0874) | (0.0875) |
|  |  |  |  |  |  |
| New Zealand | -0.0435 | -0.0705 | -0.0682 | -0.0498 | -0.0371 |
|  | (0.0438) | (0.0474) | (0.0475) | (0.0479) | (0.0481) |
|  |  |  |  |  |  |
| Female | -0.0126 | 0.0240 | 0.0323 | 0.00214 | 0.00764 |
|  | (0.0349) | (0.0389) | (0.0391) | (0.0399) | (0.0403) |
|  |  |  |  |  |  |
| *Relationship status* (Base: Single) | | | | | |
| - In a Relationship |  | -0.0273 | -0.0412 | -0.0699 | -0.0730 |
|  |  | (0.0524) | (0.0526) | (0.0530) | (0.0531) |
|  |  |  |  |  |  |
| - Married |  | 0.0573 | 0.0505 | 0.0207 | 0.0186 |
|  |  | (0.0515) | (0.0518) | (0.0523) | (0.0524) |
|  |  |  |  |  |  |
| - Child |  | 0.0362 | 0.0190 | 0.0363 | 0.0337 |
|  |  | (0.0483) | (0.0486) | (0.0494) | (0.0495) |
|  |  |  |  |  |  |
| *Education status* (Base: Did not complete high school) | | | | | |
| - High School |  | **0.203**** | **0.198**** | 0.123 | 0.135 |
|  |  | (0.0829) | (0.0831) | (0.0839) | (0.0840) |
|  |  |  |  |  |  |
| - Certificate / Diploma |  | **0.198**** | **0.189**** | 0.132 | **0.141*** |
|  |  | (0.0816) | (0.0818) | (0.0822) | (0.0824) |
|  |  |  |  |  |  |
| - Bachelor / Post-graduate |  | **0.315***** | **0.299***** | **0.188**** | **0.195**** |
|  |  | (0.0845) | (0.0850) | (0.0859) | (0.0858) |
|  |  |  |  |  |  |
| *Employment status* (Base: Full-time employed) | | | | | |
| - Part-time employed |  | -0.0566 | -0.0528 | -0.0251 | -0.0379 |
|  |  | (0.0521) | (0.0523) | (0.0527) | (0.0528) |
|  |  |  |  |  |  |
| - Retired |  | **-0.271^***^** | **-0.236^***^** | **-0.187^**^** | **-0.185^**^** |
|  |  | (0.0896) | (0.0903) | (0.0903) | (0.0908) |
|  |  |  |  |  |  |
| - No employment |  | -0.0909 | -0.0606 | -0.0465 | -0.0588 |
|  |  | (0.0559) | (0.0565) | (0.0568) | (0.0570) |
|  |  |  |  |  |  |
| *Household income* (Base: Less than $40K) | | | | | |
| - $40K - $60K |  | 0.0707 | 0.0569 | 0.0834 | **0.0931^*^** |
|  |  | (0.0556) | 0.0558 | (0.0562) | (0.0564) |
|  |  |  |  |  |  |
| - $61K - $85K |  | -0.00254 | 0.0177 | 0.0162 | 0.0213 |
|  |  | 0.0649 | (0.0651) | (0.0656) | (0.0656) |
|  |  |  |  |  |  |
| - $86K - $100K |  | -0.0233 | -0.0447 | -0.0488 | -0.0322 |
|  |  | (0.0693) | (0.0697) | (0.0699) | (0.0703) |
|  |  |  |  |  |  |
| - $100K or more |  | -0.0335 | -0.0615 | -0.0741 | -0.0780 |
|  |  | (0.0658) | (0.0666) | (0.0671) | (0.0673) |
|  |  |  |  |  |  |
| *Frequency of eating out* (Base: Seldom) | |  |  |  |  |
| - Often |  |  | **0.204***** | **0.171***** | **0.170***** |
|  |  |  | (0.0568) | (0.0571) | (0.0572) |
|  |  |  |  |  |  |
| - Normal |  |  | **0.122***** | **0.100**** | **0.108**** |
|  |  |  | (0.0460) | (0.0461) | (0.0463) |
|  |  |  |  |  |  |
| *Spending when eating out* (Base: $20 or less) | |  |  |  |  |
| - $21 – $35 |  |  | **0.126***** | **0.117***** | **0.110**** |
|  |  |  | (0.0429) | (0.0434) | (0.0435) |
|  |  |  |  |  |  |
| - $36 or more |  |  | 0.0649 | 0.0368 | 0.0278 |
|  |  |  | (0.0506) | (0.0510) | (0.0513) |
|  |  |  |  |  |  |
| *Goals* |  |  |  |  |  |
| Healthy Eating Goals |  |  |  | **0.181^***^** | **0.205^***^** |
|  |  |  |  | (0.0207) | (0.0281) |
|  |  |  |  |  |  |
| Food Economizing Goals |  |  |  | **-0.103^***^** | **-0.0765^***^** |
|  |  |  |  | (0.0191) | (0.0282) |
|  |  |  |  |  |  |
| Food Hedonism Goals |  |  |  | -0.00374 | **-0.0637^**^** |
|  |  |  |  | (0.0194) | (0.0276) |
| *Interactions* |  |  |  |  |  |
| Female*Healthy Eating Goals |  |  |  |  | -0.0600 |
|  |  |  |  |  | (0.0404) |
|  |  |  |  |  |  |
| Female*Food Economizing Goals |  |  |  |  | -0.0557 |
|  |  |  |  |  | (0.0377) |
|  |  |  |  |  |  |
| Female*Food Hedonism Goals |  |  |  |  | **0.124^***^** |
|  |  |  |  |  | (0.0386) |
|  |  |  |  |  |  |
| _cons | 1.083^***^ | 1.061^***^ | 0.950^***^ | 0.948^***^ | 0.932^***^ |
|  | (0.0503) | (0.123) | (0.126) | (0.126) | (0.126) |
| ***N*** | 996 | 885 | 885 | 885 | 885 |

*Note: Standard errors in parentheses. * p < 0.10, ** p < 0.05, *** p < 0.01. For the frequency of eating out variables often represents eating out 3 times a week or more; normal stands for eating out once or twice a week; seldom corresponds to once a month or only on special occasions. Among the employment status variables ‘No employment’ includes those who are unemployed as well as students, those not able to work, retired people and homemakers.*

**Point 1.15:** The authors could also offer further explanations of findings. For example, the authors could explore why Food Hedonism becomes non-significant in Models 3 and 4 only (at the moment, only a general comment about the strength of the effect of WTPP is offered; p.25, lines 423-424). These considerations could be offered by exploring the associations with the variables entered at different stages

**Response 1.15:**

Thank you for pointing this out. We have updated the section in which we discuss our regression results to offer further explanation. More specifically, please see the changes in pages 27 and 28 of the revised manuscript.

*For all Models, the coefficients for the healthy eating goals are consistently positive and significant with a similar magnitude throughout. Likewise, the coefficients for the food economizing variable are consistent throughout the five specifications, being significant and negative (as hypothesized). However, for the food hedonism variable we observe no statistical significance in models three and four, which is due to the addition of demographic variables, especially the age categorical variables. This suggests a closer association between age and food hedonism variable compared to food economizing and healthy eating variables. When statistically significant, we observe the effect of food hedonism goals to be negative. Overall, the effect of it on the WTPP is not as robust as the effects we observe for the healthy eating goals and the food economizing variable. In summary, healthy eating goals has the largest positive effect on the WTPP. On the other hand, food economizing and food hedonism both decrease consumers’ WTPP for healthy beverages.*

**Point 1.16:** It is also unclear why, in Model 5, the authors explored the interaction effects only for Gender and not for control variables and demographics. The explanations regarding the observed effects warrant further elaboration, too – as at the moment there is a only a mention that the relationship between hedonism and WTPP “changes for women” and it is due to “different perceptions among women” (p.26, line 455-457)

**Response 1.16:**

Thank you for pointing this out. The reason for focusing on the interaction effects of gender was driven from the literature and the past research indicating nuances among males and females with regard to health product consumption. This is explained in our literature review:

*Age and gender have been linked to consumer attitudes towards purchasing healthier food and drink options, although in different ways [38]. Some evidence suggests women are more inclined to make healthy food and drink choices, have a much higher preference for healthy products, and are more likely to purchase these regularly [39,40, Loureiro et al., 2002]. Women also report more positive attitudes and a greater willingness to purchase healthy food and beverages [41,42]. However, while some studies have identified that women are less inclined to pay a premium for organic wine [Ogbeide, Stringer & Ford, 2015], some studies found that gender plays no role in determining consumers’ WTPP for healthy/organic food products [Ali & Ali, 2020; Krystallis & Chryssohoidis, 2005]. Thus, the different contexts in which studies are conducted (i.e. food, beverages, wine) highlights the nuances in gender differences and the need to further investigate.*

References:

A.E. Ozen, A. Pons, J.A. Tur Worldwide consumption of functional foods: A systematic review. Nutr Rev. 70 (8) (2012) pp. 472-481

A. Annunziata, R. Vecchio Consumer perception of functional foods: A conjoint analysis with probiotics. Food Qual Prefer. 28 (2013) pp. 348-355

F. Bimbo, A. Bonanno, G. Nocella, R. Viscecchia, G. Nardone, B. De Devitiis, et al. Consumers’ acceptance and preferences for nutrition-modified and functional dairy products: A systematic review. Appetite. 113 (2017) pp. 141-154.

M.L. Loureiro, J.J. McCluskey, R.C. Mittelhammer Will consumers pay a premium for eco-labeled apples? J. Consum. Aff., 36 (2) (2002), pp. 203-219

O.A. Ogbeide, C. Ford, Stringer, R Consumer Willingness to Pay a Premium for the Health Benefits of Organic Wine. Mayfair Journal of Agribusiness Management. 1 (2015). pp. 1-23.

A. Tabassum, J. Ali. Factors Affecting the Consumers’ Willingness to Pay for Health and Wellness Food Products. J. of Agriculture and Food Research 2 (2020): 100076. <https://doi.org/10.1016/j.jafr.2020.100076>.

A. Krystallis, G. Chryssohoidis Consumers' willingness to pay for organic food: factors that affect it and variation per organic product type Br. Food J., 107 (5) (2005), pp. 320-343

In conducting our analysis, we did not find gender to be directly related to WTPP, however, further reinvestigating gender as a moderator variable in the relationship among motivational goals and WTPP revealed some interesting interaction effects (as we report in our results and discussion).

*Finally, the extant literature suggests women are more inclined to make healthy food and drink choices and to purchase more regularly [39,40, Loureiro et al., 2002]. Our study expands on the body of knowledge by examining if the relationships among motivational goals (healthy eating, economizing, hedonism) and WTPP are moderated by gender differences. Results of the interaction effects found gender to have no significant moderating effects on the relationship between health goals and WTPP, or economizing goals and WTPP. However, there is evidence of moderation between hedonism goals and WTPP, suggesting different perceptions among women as to the association between healthy products and hedonistic consumption. The relationship between food hedonism and WTPP is negative for men, but switches sign when it comes to women. Men that are high on the food hedonism scale are less likely to pay a premium for healthy beverages. Compared to men, however, women with a similar food hedonism scores are willing to pay more of a premium.*

**Point 1.17:** Related to the above, other effects should be discussed further. For example, although spending when eating out matters, it is interesting to note that this is true only for the $21-$35 vs. $20 comparison, but not for the $36 vs. $20 comparison, which seems counter-intuitive. The same applies for household income, where only the $40-$60k v < $40k comparison is significant, but none of the other income groups

**Response 1.17:**

Thank you for pointing this out. We have updated the Discussion section in which we discuss the eating out spending and income results to offer further explanation.

*Most of the control variables in our regression analysis have expected signs and statistical significance. Consumers spending $21-$35 when eating out are willing to pay more for healthy beverages. Our explanation for this is that an expenditure amount of $36 +, the purchase expands beyond a standard meal and drink and most likely includes wine or desert, in which case healthy eating goals are can be superseded by enjoyment of the dining experience.*

*WTPP for healthy beverages* *increases with eating out frequency. Compared to those who seldom eat out (once a month or on special occasions), consumers who eat out regularly/normal (1-2 week) and often 3 or more times a week) are more willing to pay a premium for a healthy beverage. The premium price they are willing to pay also increases with their eating out frequency; those who eat out more often are willing to pay a larger premium. Similarly, WTPP for healthy beverages decreases with age, with the youngest cohort (aged 18-24) willing to pay the largest premium for healthy beverages. We also observe a positive association with higher education and our dependent variable, but only for respondents with a Bachelor’s degree or higher. Other control variables do not show any statistical significance, with one exception – the coefficient for those whose employment status is ‘retired’ is consistently negative and significant. The results for the income variables are also worth mentioning. Contrary to the previous findings in the literature, our results suggest almost no association between income and WTPP, except for a marginally significant effect for the $40K - $60K group, compared to those who make less than $40K. However, this result is in accord with the findings for the consumers spending when eating out. Together, these results might suggest an inverse-U shaped relationship between expenditure when eating out, income and willingness to pay premium for healthy beverages.*

**Point 1.18:** The Discussion seems to focus much on summarising the outcomes rather than elaborating on additional insight (e.g., p.31). As mentioned above, some conclusions are based only on descriptive statistics and thus should be formulated more tentatively. Other arguments (e.g., about potential differences between AUS and NZ, lines 584-588 on p.33) are only hinted at, too. Relatedly, some arguments offered in regards to limitations should be explored further, for example in terms of sample size and the use of more explicit criteria for categorising healthy beverages, which has been previously mentioned as a strategic choice of the study

**Response 1.18:**

Thank you for your feedback on the Discussion. Based on your comments, the ‘Discussion’ section and the ‘Conclusion, limitations, and future research directions’ have now been completely re-drafted. Please refer to the new revisions highlighted in the manuscript.

**Point 1.19:** p.8, line 185: Define what hedonistic approach towards food is as soon as you introduce this construct

Response 1.19:

Thank you, the revised Literature Review section of the manuscript now addresses this point.

**Point 1.20:** p.14, line 300: It is not clear why the authors mention that the count data “are likely” to be skewed; after all, the authors have these data and thus should be reporting on them more specifically.

**Response 1.20:**

Thank you for pointing this out. The wording you mention refers to a general count data setup. We now realise that the sentence might be confusing to the readers and have removed this from the manuscript.

**Point 1.21:**p.17: The authors may offer additional detail about coding of answers, for example whether multiple and independent assessors were required to ensure consistency in terms of responses to open questions

Response 1.21:

Thank you for this suggestion. We have considered your point but believe this is not applicable to our case. As we explained in our response to Point 1.13, the content of responses to the open questions were basic and straightforward recounts. Qualitative analysis of the open-ended question is not required due to simple responses (e.g water, tea, no sugar, organic). It is sufficient to report these based on frequency as we have done in Table 2 and 3. We also reiterate that while this information regarding consumers’ perceptions of what is healthy (and why) is a component of our study, it is not the major objective of the research which is to examine the factors driving willingness to pay a premium price.

**Point 1.22:** On the regression table, I wonder whether the authors could enter information about percentages (as explained in lines 428-436) so that the reader can more easily appraise the size of the effect of each predictor.

**Response 1.22:**

Thank you, this is an excellent suggestion. As per your recommendation, for each statistically significant estimate in our regression table we now include the corresponding percentages (see Table 5)

**Reviewer 2:**

**Point 2.1:** This is an interesting and useful study. The paper is well written and results are clearly presented, but the discussion conclusions sections lack some clarity. This study should be of interest to the readers of this journal and I support the publication of the paper with some revisions to address some of the major comments below.

**Response 2.1**:

Thank you for your support. We have addressed all comments presented below and made substantial revisions to the manuscript.

**Point 2.2:** First, the study will be most useful for NZ and Au policy makers and hospitality industries and less applicable to other parts of the world, which is recognized by the authors as a limitation. How do you believe your study areas are different from other areas? What features may be unique? If you want readers to understand how generalizable the results are or which ones are and which are not, more discussion would be helpful

**Response 2.2:**

Thank you for raising this issue. The manuscript has been significantly reworked to strengthen its originality and contribution. The Introduction and Literature Review have been substantially revised with a new narrative that strengthens the study’s position and contribution. These changes also follow through to the ‘Discussion’ and the ‘Conclusion, limitations and future research directions’ where the contribution and implications of the study are now more explicit. Please see highlighted content in revised manuscript.

Drawing on the marketing and food literature, our study hypothesized that 1) Women will have a significantly higher willingness to pay a price premium for healthy beverage products compared to men; and, 2) Older consumers will have a significantly higher willingness to pay a price premium for healthy beverage products as compared to younger consumers. Interestingly, neither of these hypotheses could be supported. While some previous studies suggest women are likely to have a higher preference for healthy products, and are more likely to purchase these regularly [36,37,44], our findings are more consistent with others that have found no role of gender determining consumers’ WTPP for healthy/organic food products [38,39]. Moreover, in the context of organic wines some research suggests women are less inclined to pay a premium [51]. Thus, the effects of gender in determining WTPP for health products remains inconclusive, and marketers should focus more on motivational and behavioral characteristics. A similar case can be made for age groups. Our findings that younger (18–24 year-old) consumers have a higher willingness to pay a premium for healthy beverages than older consumers are in contrast to previous literature [47,48]. This suggests that younger segments of the market now have a higher awareness of diet, health and nutrition, and again emphasizing that psychographic variables and motivations goals are far more pertinent in understanding purchase behaviors than age or gender.

36.	Annunziata A, Vecchio R. Consumer perception of functional foods: A conjoint analysis with probiotics. Food Qual Prefer. 2013;28: 348-355

37.	Loureiro ML, McCluskey JJ, Mittelhammer RC. Will consumers pay a premium for eco-labeled apples? J Consum Aff. 2002;36: 203-219.

44.	Bimbo F, Bonanno A, Nocella G, Viscecchia R, Nardone G, De Devitiis B, et al. Consumers’ acceptance and preferences for nutrition-modified and functional dairy products: A systematic review. Appetite. 2017;113: 141-154.

**Point 2.3:** Second, although their literature review supports a positive correlation of income with WTPP, hence their H3, the authors spend no time providing any intuition as to why they found no support in their data for H3. I think it is important to dedicate some discussion space to this result. Similarly, H1 and H2 also need more discussion to understand why the data in this study contradicts prior research. On lines 534 and 535 there is an explanation about the gender effects regarding the different perceptions among women but it is left to the reader to guess what those different perceptions are in fact. This explanation is repetitive (lines 455, 456, 457) and the discussion offers no more insight than results section

**Response 2.3:**

Thank you raising this issue. We have now expanded on the findings and discussion in regard to the hypotheses tests H1, H2 and H3. Please refer to highlighted content of the Discussion as this now addresses these points raised.

**Point 2.4:** Third, it would be good to see the policy outcomes spelled out a bit more clearly and broadly (based on the three hypotheses for which they did find support).

***Response 2.4:***

The manuscript has been revised and the policy implications made more explicit. See ‘Conclusion, limitations, and future research directions’

*.* The widespread distribution of high calorie sugar-sweetened beverages in the hospitality industry continues to have negative effects on public health [19]. The hospitality industry and foodservice sector can play an important role in the development and supply of healthier options, nudging consumers to choose healthy. Understanding the healthy beverage sector from the consumers’ perspectives provides valuable insights to the hospitality industry and business operators.

Regarding implications for policy and practice, our study highlights the need for a collaborative effort among government, beverage manufacturers, and the hospitality sector in increasing the variety and supply of healthy product options. Evidence of strong consumer demand and willingness to pay should drive innovation in new product development and distribution, bringing products that are healthy, taste good, and reasonable priced to hospitality consumers. These measures will address the reported barriers to purchasing, as revealed in our study, regarding the limited availability of healthy beverage products sold in hospitality venues. In addition, consumer skepticism around ‘health’ claims of products should also be addressed by providing validated information about the product’s attributes. Beverages should include Front of Package (FoP) nutritional labeling, such as certified Health Star Ratings, as this information is perceived to be more credible than marketing communication [30]. The strong links between consumers’ healthy eating goals and WTPP suggests a need for Government-led social marketing initiatives that influence people’s motivations and attitudes toward health and wellbeing. These cognitive factors are strong drivers of intention to purchase healthier products and encouraging health conscious (as opposed to hedonistic) decisions.

In terms of limitations, data for this study was collected from hospitality industry consumers in Australia and New Zealand. Our insights on what consumers determine to be healthy beverages and their attributes are based on descriptive analysis of the sample responses, thus, the generalizability of these findings to the broader population needs further testing. Nevertheless, it is important to note that our sampling design was representative of the broader population for each country. We encourage future studies to collect data from different countries and examine cross-cultural/country differences among consumers’ purchase behaviors. Larger samples could also be collected to allow for a multigroup comparison between respondents across various psychological and demographic factors. In addition, an in depth understanding of how consumers make judgements regarding the healthiness of a beverage, and what information they use to make these assessments, is warranted to understand consumers’ knowledge of health and nutrition and its impact on consumption.

As noted in this study the definition of ‘healthy’ was not stipulated in to order to gain insights through respondents’ self-evaluations. Future research aiming to examine objective measures of ‘healthiness’ could use more explicit criteria based on the product’s quantifiable attributes, ingredients and nutritional profile (e.g. amount of sugar, calories, fat etc.). Despite these limitations, this study presents new contributions to the body of knowledge on consumers’ attitudes, motivations, and purchase behaviors concerning healthy beverages.

**Point 2.5:** Fourth, there are other papers that aren’t mentioned in the paper that the authors might consider.

Ali, Tabassum, and Jabir Ali. “Factors Affecting the Consumers’ Willingness to Pay for Health and Wellness Food Products.” Journal of Agriculture and Food Research 2 (2020): 100076. [https://doi.org/10.1016/j.jafr.2020.100076](https://protect-au.mimecast.com/s/oSxECjZ1qOCPDBnkfjXJ_w?domain=doi.org).

A. Krystallis, G. Chryssohoidis
Consumers' willingness to pay for organic food: factors that affect it and variation per organic product type Br. Food J., 107 (5) (2005), pp. 320-343

S. Gao Effects of additional quality attributes on consumer willingness to pay for food labels
Ann. Phys. (2007), pp. 1-133

M.L. Loureiro, J.J. McCluskey, R.C. Mittelhammer Will consumers pay a premium for eco-labeled apples? J. Consum. Aff., 36 (2) (2002), pp. 203-219

Ogbeide, Osadebamwen. (2015). Consumer Willingness to Pay a Premium for the Health Benefits of Organic Wine. Mayfair Journal of Agribusiness Management. 1. 1-23.

**Response 2.5**:

Thank you for suggesting these references. These studies have been included in our literature review and discussion

31.	Bates S, Reeve B, Trevena, H. A narrative review of online food delivery in Australia: Challenges and opportunities for public health nutrition policy. Public Health Nutr. 2020;1–11. doi: 10.1017/S1368980020000701.

37.	Loureiro ML, McCluskey JJ, Mittelhammer RC. Will consumers pay a premium for eco-labeled apples? J Consum Aff. 2002;36: 203-219.

38.	Ali T, Ali, J. Factors affecting the consumers’ willingness to pay for health and wellness food products. J Agric Food Res. 2020;2: 100076. doi: 10.1016/j.jafr.2020.100076.

39.	Krystallis A, Chryssohoidis G. Consumers' willingness to pay for organic food: Factors that affect it and variation per organic product type. Br Food J. 2005;107: 320-343.

40.	Gao Z, Schroeder TC. Effects of label information on consumer willingness-to-pay for food attributes. Am J Agric Econ. 2009;91: 795-809.

51.	Ogbeide O. Consumer willingness to pay a premium for the health benefits of organic wine. Mayfair Journal of Agribusiness Management. 2015;1: 1-23.

**Point 2.6**

Minor editing suggestions – typos and grammatical errors
Line 32 – finds instead of find- ok
Line 67 – there are also growing concerns - corrected
Line 144 “…established than younger people’s” - corrected
Line 265 “ through” is unnecessary - corrected
Line 354 – 66% of respondents - corrected
Line 356 – almost 79% of respondents spent - corrected
Line 357 – consistent with Australian - corrected
Line 493 – which have established health benefits - corrected
Line 549 – sugar sweetened beverages- corrected
Line 550 - that remain prevalent- corrected
Line 552 – sugar sweetened drinks - corrected
Line 575 – food service sector- We have sue the term foodservice sector which is common used to represent this sector

**Response 2.6:**

Thank you for identifying these corrections. The revised manuscript has addressed all of these corrections. Due to the major changes made to manuscript, some of these sentences have been deleted.
